# Impact of COVID-19 pandemic on the mental health of university students in the United Arab Emirates: a cross-sectional study

**DOI:** 10.1186/s40359-022-00986-3

**Published:** 2022-12-16

**Authors:** Anamika Vajpeyi Misra, Heba M. Mamdouh, Anita Dani, Vivienne Mitchell, Hamid Y. Hussain, Gamal M. Ibrahim, Wafa K. Alnakhi

**Affiliations:** 1 Department of Health Sciences- Social Work Program, Higher Colleges of Technology, Abu Dhabi, UAE; 2 General Academic Requirement (General Studies), Higher Colleges of Technology, Abu Dhabi, UAE; 3 Department of Data Analysis, Research and Studies, Dubai Health Authority, Dubai, UAE; 4 Department of Family Health, High Institute of Public Health, Alexandria University, Alexandria, Egypt; 5 Mohammed Bin Rashid University of Medicine and Health Sciences, Dubai, UAE

**Keywords:** COVID–19 pandemic, Psychological impact of Covid-19, Mental health, University students, United Arab Emirates

## Abstract

**Background:**

The COVID-19 pandemic had a significant impact on the mental well-being of students worldwide. There is a scarcity of information on the mental health impact of the COVID-19 pandemic on university students in the United Arab Emirates (UAE). This study aimed to investigate the mental health impact of the COVID-19, including depression, anxiety and resilience among a sample of university students in the UAE.

**Methods:**

A cross-sectional study using an online survey was conducted from September to November 2021. The patient health questionnaire (PHQ-9), generalized anxiety disorder-7 (GAD-7) and Connor–Davidson Resilience Scale (CD-RISC-10) were used to assess depression, anxiety, and resilience. The COVID-19 impact was assessed using a list of questions.

**Results:**

Only, 798 students completed the survey and were analyzed for this study. Overall, 74.8% of the students were females, 91.2% were never married, and 66.3% were UAE-nationals. Based on PHQ-9 and GAD-7 cut-off scores (≥ 10), four out of ten of the students self-reported moderate to severe depression (40.9%) and anxiety (39.1%). Significantly higher mean PHQ-9 and GAD-7 scores were found among students who were impacted by COVID-19 than those non-impacted (mean PHQ-9 = 9.51 ± 6.39 and 6.80 ± 6.34; *p* = 0.001, respectively) and (mean GAD-7 = 9.03 ± 6.00 and 8.54 ± 6.02; respectively, *p* < 0.001). Female students who were impacted by COVID-19 had statistically significant higher depression and anxiety scores (mean PHQ-9 of 9.14 ± 5.86 vs. 6.83 ± 6.25, respectively; *p* < 0.001) than the non-impacted females (mean GAD-7 of 9.57 ± 6.32 vs. 5.15 ± 3.88, respectively; *p* = 0.005). Never married students had significantly higher PHQ-9 and GAD-7 scores than ever-married (9.31 ± 6.37 vs. 6.93 ± 5.47, *P* = 0.003) and (8.89 ± 6.11 vs. 7.13 ± 5.49, respectively; *p* = 0.017).

**Conclusions:**

The results of this study demonstrate that the COVID-19 pandemic has negatively impacted the mental health of this sample of university students in terms of depression and anxiety. The results highlight the need to adopt culturally appropriate interventions for university students and focus on vulnerable groups.

**Supplementary Information:**

The online version contains supplementary material available at 10.1186/s40359-022-00986-3.

## Introduction

In March 2020, the World Health Organization declared the coronavirus disease 2019 (COVID-19) a world pandemic status [1]. The pattern of the virus has affected many aspects including physical wellbeing, psychosocial life, and the local and global economy [2]. The high morbidity and mortality rates and the ambiguity around the ongoing pandemic have brought up many mental sufferings for a large proportion of people worldwide [3]. In addition, the unprecedented public health interventions that were implemented across the globe, including the United Arab Emirates (UAE) caused a wide range of psychosocial impacts [4]. The societal effects of the COVID-19 pandemic are so pervasive—and yet vary so tremendously according to individual and contextual factors—that global characterization regarding its psychological impact is likely impossible [2, 5, 6].

Several studies have looked at the impact of epidemics on population mental health over the last few decades, and they have reported a wide range of psychological impacts [7–10]. Around the world, published research on the impact of the COVID-19 pandemic on mental health revealed that the pandemic is linked to an increase in the rates of depression, anxiety, stress and sleep disturbance among various population groups [11–17]. Research endorsed that universal pandemics can endanger one’s mental well-being since only some people are resilient to change in their environment and able to seek out psychological assistance when needed. Whilst others may emphasize on the physical aspect of themselves during the pandemic time rather than their mental well-being [18]. Psychologists define resilience as the process of adapting well in the face of adversity, trauma, tragedy, threats, or significant sources of stress—such as family and relationship problems [19]. People's reactions to crises vary, yet coping strategies to manage such situations require more investigation.

It is clear that the burden of mental health adverse outcomes of the pandemic is not equally shared. Indeed, a substantially greater risk accrues to those facing ongoing stressors, such as job loss, economic distress, occupational stress, responsibilities, social isolation, interpersonal loss, and virus exposure. Moreover, specific dispositional vulnerabilities or diatheses (such as internalizing tendencies or fears of contamination) could interact with the stress and may substantially increase the risk [20].

University students’ mental health issues are not well recognized and infrequently addressed. Students at universities are often at a vulnerable age (between adolescence and early adulthood), making them sensitive to mental illnesses [21]. Research revealed that student status was associated with a higher frequency of depressive and anxiety symptoms, perceived stress, and suicidal thoughts [21]. Blanco et al. estimated in their early research that half of the college-age people they surveyed had a mental health issue [22]. Literature showed that although the physical implications of COVID-19 were milder on young adults, their mental health was negatively impacted by the pandemic [23]. Reduced socialization along with the quarantine protocols due to COVID-19 resulted in worsened mood status and increased anxiety during the pandemic [23]. Patwary et al. 2022 found in their study that more than three in four students experienced clinically significant anxiety levels during the early stages of the COVID-19 pandemic. [24]. The mental health of young people has been a concern in the UAE, where a published study of the mental health of university students in the UAE (as screened by PHQ-9) found that the prevalence of depression among university students was estimated to be 22.2% [25]. In addition, a previous pre-pandemic study from the UAE revealed significant levels of anxiety among young adults, making this group especially prone to mental health issues [26].

Higher colleges of Technology (HCT), founded in 1988, is one of the largest applied higher education institutions, with 16 campuses across the UAE. Currently, there are 21,572 students enrolled in the HCT under 72 programs [27]. During the COVID-19 pandemic, HCT remained agile and swiftly moved to the online classes and assessments, then continued the hybrid learning model of education.

Despite excellent precautionary, preventative and therapeutic healthcare measures, being put in place by the UAE government, and the lower COVID-19 infection rates than the global average (8.12%), the psychological impact of COVID-19 on the UAE population should not be overlooked [25, 26]. Information about the influence of COVID-19 on the mental health of the different sectors of the UAE population is limited [28]. Few published research pointed to a high prevalence of anxiety, depression, and stress among the general public [29], healthcare workers [30, 31], and the elder population [32]. However, the mental health effects on university students within the UAE are inadequately addressed. Given these situations, it is important to investigate the university students’ mental health during the COVID- pandemic to inform the possible interventions. Therefore, the current study aimed to address a number of existing gaps including the COVID-19 impact on mental health, in particular depressive and anxiety symptoms, as well as to assess the resilience of a sample of HCT university students in the UAE during the COVID-19 pandemic. It also investigated the effect of some socio-demographic characteristics and the COVID- 19 impact on the mental health of the sampled students.

## Materials and methods

### Study population, design and setting

A cross-sectional study was conducted among a sample of students who were enrolled in the undergraduate and postgraduate programs of the HCT university across the UAE. A structured self-administered questionnaire was used for data collection in the current study. Participants were recruited via announcements through the email network of the HTC University. The data collection took place online from September to November 2021. The responses were extracted using an electronic survey via the google survey tool (Google Forms). Participants were asked for consent approval before participation. The median completion time for the survey was 9 min. Based on the Raosoft calculator for sample size estimation, the minimum required sample for this study was 378 with a confidence interval of 95.0 and 0.5 margin of error [33]. Out of the total survey sent, 819 students voluntarily responded with a response rate of 43%. Only, 798 students fully completed the survey and were analyzed for this study.

### Variables and measures

The questionnaire included socio-demographic demographics, COVID-19 -related Items, 9-item patient health questionnaire (PHQ-9), 7-item generalized anxiety disorder (GAD-7) scale and the 10-items Connor–Davidson Resilience Scale (CD-RISC-10). The socio-demographics included gender, age groups, nationality, marital status, working status (Currently employed or not employed) and Emirate of residence within the UAE. Nationality was dichotomized to UAE nationals and non-UAE nationals. Marital status was grouped into ever-married that included married and divorced/widowed or single/ never married participants.

The impact of Covid-19 on the participants was assessed using an outcome variable (the COVID-19 impact). The variable was dichotomized into those who were impacted by COVID-19 or not impacted by COVID-19. Seven questions in the survey assessed if the respondents were impacted by COVID-19 in some way or another. “Impacted by COVID-19” was defined if the participants answered “yes”, they were diagnosed with COVID-19 themselves or a close family/ friend, witnessed a COVID-19 related death or had high exposure to COVID-19 at the workplace in the past year preceding the survey. The respondents who answered no to all of the seven questions were grouped in the category of “not impacted by COVID-19”.

### Mental health assessment scales

#### The Patient Health Questionnaire-9 (PHQ-9)

The PHQ-9 is a 9-item depression assessment module adopted from the full PHQ. The PHQ-9 has been previously recognized as a valid and reliable instrument for screening of depression in the general population and in university context [34–36]. It consists of nine questions probing the frequency of depressive symptoms over the past 2 weeks. Responses ranged from 0 to 3 (0 = not at all, 1 = several days, 2 = more than half the days, 3 = nearly every day). Total scores, obtained by summing the responses to each item, range from 0 to 27. Cut-off scores adopted in the present study included scores of ≤ 9 and ≥ 10 that suggest minimal to mild depression and moderate to severe depression on the PHQ-9, respectively [35]. The reliability of the scale among the current sample was excellent (α = 0.876).

#### The generalized anxiety disorder-7 (GAD-7)

The Gad-7 is widely used as a self-reporting scale to assess the symptoms of anxiety. It consists of 7-Items that measures anxiety over the past 2 weeks. Items are rated on a 4-point Likert-type scale (0 = not at all, 1 = several days, 2 = more than half the days, 3 = nearly every day). The GAD-7 score is calculated by assigning scores of 0, 1, 2, and 3, then adding together the scores for the seven questions. GAD-7 total score for the seven items ranges from 0 to 21. Cut-off scores of ≤ 9 and ≥ 10 are considered minimal to mild and moderate to severe levels of anxiety on the GAD-7, respectively [37, 38]. The reliability of the scale among the current sample was excellent (α = 0.906).

#### Connor–Davidson Resilience Scale (CD-RISC-10)

CD-RISC-10 is a widely used self-reported questionnaire [39, 40]. It consists of 10-items to assess the population’s resilience levels or their ability to tolerate and overcome adverse situations such as illness, pressure, and failure. Each item is rated on a 5-point Likert scale (0 = not true at all, 1 = rarely true, 2 = sometimes true, 3 = often true, and 4 = true nearly all of the time), with a higher total score indicates greater resilience. Due to the lack of a recognized cut-off point, resilience scores were categorized into high resilience (score ≥ 33) and normal or low resilience (score ≤ 32) [39, 40]. The reliability of the scale among the current sample was excellent (α = 0.893).

#### Estimating the prevalence and the levels of depression and anxiety

Prevalence rates of depression and anxiety were determined using cut-off points based on PHQ-9 and GAD-7 scales validation [34, 37]. In the current study, depression was defined as a total score of (≥ 10) in the PHQ-9 instrument, indicating a case of moderate to severe depression. Anxiety was defined using the GAD-7 instrument with a total score of (≥ 10), indicating a case of moderate to severe anxiety. The prevalence of depression or anxiety was estimated by dividing the number of students who exceeded the cut-off score by the total number of students who responded.

### Statistical analysis

Data coding, data cleaning, and analysis have been carried out by using IBM SPSS (Version 22.0, IBM SPSS, IBM Corp, USA). Cronbach’s alpha coefficients were calculated to indicate scale reliability. Outliers were observed on the PHQ-9 scale, indicating that only four male and two female respondents had severe depression. Descriptive statistics, including means, standard deviations (+ SD) and percentages were used to illustrate participants’ demographics. The normal distribution of data was verified using box plots and histograms. Complete case analysis was considered in this study, then 12 missing cases with responses were excluded from the statistical analysis. The equality of variances was checked using Levene’s test. Independent sample T-test was used to compare the mean scores between the participant COVID-19 impact category and mean scores of the three scales (depression, anxiety and resilience). Participants’ anxiety, depression and resilience mean scores were compared with demographics characteristics using independent-samples t-test, one-way analysis of variance (ANOVA). An independent samples t-test was used to compare the mean scores of the three psychometric scales (anxiety, depression, and resilience scales) between different socio-demographic groups and between the COVID-19 impact categories, separately.

Univariate analysis of variance (ANNOVA) was used to examine if the mean score of the three psychometric scales (anxiety, depression and resilience scales) were different between the impacted by COVID-19 and non-impacted and participants’ gender. Analysis of between-subject effects was run to examine the effect of those categorized as impacted by COVID-19 and those not-impacted students revealed insignificant differences for the mean scores of GAD-7, PHQ 9 and CD-RISC 10 scales. The statistical significance of ≤ 0.05 was considered in the study, with 95% confidence intervals.

### Ethical approval and consent

The study was approved by the Higher College of Technology research ethics review board. Participants gave online written consent to participate in the study prior to starting the survey.

## Results

Table 1 shows the socio-demographic characteristics of the participants. It reveals that 74.8% of the students were females, and the majority (91.2%) were single/ never married. Most of the participants (66.3%) were UAE-nationals. As for the Emirate of residence, 38.1% reported living in Abu Dhabi city. The students’ age ranged from 16 to 41 years, with the highest proportion in the age group of 19 to 25 years (63.5%). Overall, 65.5% of the students stated they were currently not employed.

**Table 1:** Sociodemographic characteristics of the participants (N = 798)

Characteristic	Number (%)
*Gender*	
Males	196 (24.6)
Females	597 (74.8)
*Marital status*	
Single/ Never married	728 (91.2)
Ever married	67 (8.8)
*Current age (in years)*	
16–18	258 (32.3)
19–25	507 (63.5)
26–33	21 (2.6)
34–41	12 (1.5)
*Nationality*	
UAE-Nationals	529 (66.3)
Non-UAE-Nationals	269 (33.7)
*Employment status*	
Currently not-employed	523 (65.5)
Currently employed	275 (34.5)
*Residence (by emirate)*	
Abu Dhabi & Western Region	304 (38.1)
Dubai	142 (17.8)
North Emirates	352 (44.1)
Total	798 (100.0)

**Fig. 1:**
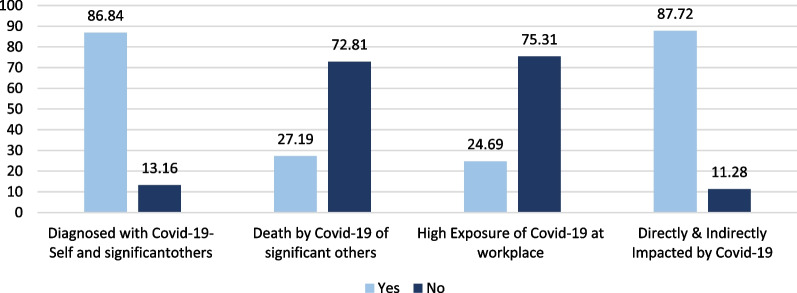
Distribution of the participants by COVID-19 related questions

The distribution of the participants by COVID-19 related questions is clarified in Fig. 1. It was revealed that the majority of the participants (88.7%) were classified as impacted by COVID-19 (as per the COVID-19 impact questions). The vast majority of students reported they were diagnosed with COVID-19 themselves or a significant relatives/ friends (86.8%). Additionally, 27.2% of students stated they knew some close relatives/ friends who died from COVID-19 or its complications.

**Fig. 2:**
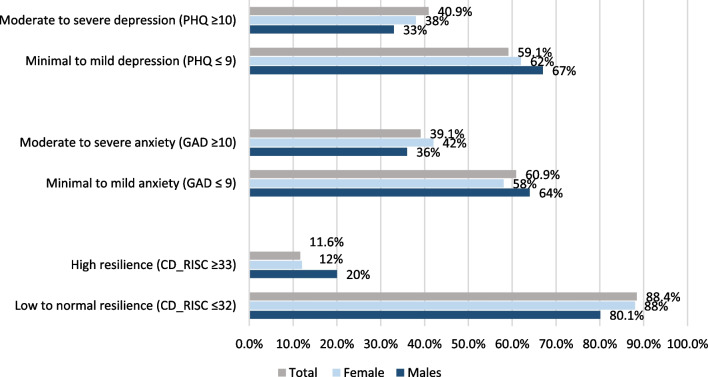
Prevalence of depression, anxiety and resilience by gender among the participants

Prevalence estimates of depression, anxiety and resilience (as measured by PHQ- 9, GAD-7 9, and CD-RISC-10 cut-off scores) by gender among the participants were summarized in Fig. 2. Overall, four out of ten of the participants had moderate and severe depression and anxiety (40.9% and 39.1%, respectively). A slightly higher proportion of females had moderate to severe depression and anxiety than males. It can be seen that males had higher resilience (12%) than females (9%). Prevalence estimates of depression, anxiety, and resilience by COVID-19 impact among the participants are shown in Table 2. Based on PHQ-9 cut-off scores (≥ 10), the self-reported prevalence of moderate to severe depression symptoms was 40.9%, and it was higher in students who were categorized as impacted by COVID-19 (43.8%) than those who were not impacted (17.8%). Based on GAD-7 cut-off scores (≥ 10), the self-reported prevalence of moderate to severe anxiety was 39.1%. Students with moderate to severe anxiety symptoms categorized as impacted by COVID-19 had higher scores (40.1%) than those who were not impacted (31.8%). Few students (11.5%) self-reported high levels of resilience (based on CD-RISC-10 score ≥ 33).

**Table 2:** Prevalence estimates of depression, anxiety and resilience (as measured by PHQ 9, GAD-7 and CD-RISC 10 scales) among the participants by covid-19 impact

Psychometric property	Impacted by Covid-19 N (%)	Not Impacted by Covid-19 N (%)	Total sample N (%)
*Depression as measured by PHQ-9*			
Minimal to mild depression (score ≤ 9)	398 (56.2)	74 (82.2)	472 (59.1)
Moderate to severe depression (score ≥ 10)	310 (43.8)	16 (17.8)	326 (40.9)
*Anxiety as measured by GAD-7*			
Minimal to mild anxiety (score ≤ 9)	424 (59.9)	62 (68.9)	486 (60.9)
Moderate to severe anxiety (Score ≥ 10)	284 (40.1)	28 (31.8)	312 (39.1)
*Resilience as measured by CD-RISC-10*			
Low to normal resilience (≤ 32)	631 (89.1)	75 (83.3)	706 (88.5)
High resilience (score ≥ 33)	77 (10.9)	15 (16.7)	92 (11.5)
Total	708 (100)	90 (100)	798 (100)

*PHQ-9* patient health questionnaire, *GAD-7* generalized anxiety disorder-7, *CD-RISC-10* Connor–Davidson Resilience Scale

Independent sample t-test for the comparison between the mean scores (± SD) of the psychometric scales by COVID-19 impact is presented in Table 3. Notably, the total mean scores (± SD) of all the three psychometric scales used were below the assumed cut-off threshold of moderate to severe depression (9.10 ± 6.33) or moderate to severe anxiety (8.78 ± 6.07), and high resilience level (21.46 ± 8.80) for the participating students. Significantly higher mean PHQ-9 and GAD-7 scores were found among students who were impacted by COVID-19 than those non-impacted. No statistically significant difference was detected in the mean CD-RISC-10 scores for those who were impacted by COVID-19 and those who were non-impacted.

**Table 3:** Independent sample T-test comparing the mean scores (± SD) of the PHQ- 9, GAD-7 and CD-RISC -10 psychometric scales by COVID-19 impact

Scale	Impacted by COVID-19	Not-impacted by COVID-19	T value	*p* value*	Total score
	Mean scores (± SD)				
PHQ-9	9.51 (6.39)	6.80 (6.34)	− 5.28	< 0.001**	9.10 (6.33)
GAD-7	9.03 (6.00)	8.54 (6.02)	− 3.30	< 0.001**	8.78 (6.07)
CD-RISC-10	21.42 (8.51)	21.75 (10.85)	0.33	0.041*	21.46 (8.80)

*PHQ-9* patient health questionnaire, *GAD-7* generalized anxiety disorder-7, *CD-RISC-10* Connor–Davidson Resilience Scale

*Significant at *p* < 0.05

** Significant at *p* < 0.001

Independent sample t-test was used to compare the mean scores (± SD) for the three psychometric scales by socio-demographic characteristics (as shown in Table 4). The mean scores of the three psychometric scales (PHQ-9, GAD-7, and CD-RISC-10) were insignificantly different between male and female participants (*p* > 0.05). Participants of UAE-nationality had significantly higher mean scores ± SD for PHQ-9 than their non-national counterparts (9.63 ± 6.51 vs. 7.92 ± 5.81, respectively, *p* = 0.001*). As for the employment status, currently non-employed participants had significantly higher CD-RISC-10 scores than the currently employed ones (22.01 ± 8.60 and 20.44 ± 9.10, respectively; *p* = 0.018*). For the marital status single/never married participants had significantly higher PHQ-9 and GAD-7 scores than ever-married (9.31 ± 6.37 and 6.93 ± 5.47, *p* = 0.003) and (8.89 ± 6.11 and 7.13 ± 5.49, respectively; *p* = 0.017*).

**Table 4:** Independent sample T-test comparing the mean scores (± SD) of the PHQ 9 & GAD-7 and CD-RISC 10 psychometric scales by demographic characteristics

Scale	Mean scores (± SD)		T value	*p* value*
Characteristic	Male	Female		
PHQ-9	9.13 (6.26)	9.01 (6.15)	− 0.230	0.821
GAD-7	8.35 (6.44)	8.90 (5.93)	− 1.10	0.273
CD-RISC-10	21.59 (9.07)	21.40 (8.72)	0.25	0.802
Characteristic	UAE-national	Non-national		
PHQ-9	9.63 (6.51)	7.92 (5.81)	3.487	0.001*
GAD-7	8.99 (6.12)	8.40 (5.95)	1.26	0.28
CD-RISC-10	21.78 (8.54)	20.85 (9.34)	1.339	0.18
Characteristic	Currently employed	Non-employed		
PHQ-9	8.70 (6.22)	9.31 (6.39)	1.29	0.61
GAD-7	8.59 (6.00)	8.89 (6.12)	0.661	0.50
CD-RISC-10	20.44 (9.10)	22.01 (8.60)	2.37	0.018*
Characteristic	Ever married	Single/never married		
PHQ-9	6.93 (5.47)	9.31 (6.37)	3.00	0.003**
GAD-7	7.13 (5.49)	8.89 (6.11)	2.39	0.017*
CD-RISC-10	21.40 (9.09)	21.47 (8.78)	0.67	0.94

*PHQ-9* patient health questionnaire, *GAD-7* generalized anxiety disorder-7, *CD-RISC-10* Connor–Davidson Resilience Scale

*Significant at *p* < 0.05

** Significant at *p* < 0.001

The interaction between the effects of COVID-19 impact and gender on the mean scores (± SD) of PHQ 9, GAD-7, and CD-RISC 10 psychometric scales were examined using a two-way ANNOVA test are shown in Table 5. There was a statistically significant interaction between the effects of gender and COVID-19 impact on both depression and anxiety scores. In particular, females who were categorized as impacted by COVID-19 (interaction term) had a significantly higher mean PHQ-9 score ± SD than those who were not impacted (9.14 ± 5.86 vs. 6.83 ± 6.25, respectively; *p* < 0.001). Similarly, females who were impacted by COVID-19 had a significantly higher GAD-7 score than the ones who were non-impacted impacted (9.57 ± 6.32 vs. 5.15 ± 3.88, respectively; *p* = 0.005). Resilience mean scores were almost similar in females who were impacted by COVID and those who were not. No significant differences were detected in the mean scores of any of the mental health scales studied for male participants by COVID-19 impact. The interaction between the effects of COVID-19 impact and marital status and nationality group on mean scores of PHQ 9, GAD-7, and CD-RISC 10 psychometric scales were non-significant (Additional file 1: Appendix 1).

**Table 5:** Two-way ANNOVA for comparing the differences in mean scores of PHQ 9, GAD-7 & and CD-RISC 10 scales among the participants by COVID-19 impact and gender

Scale	Impacted by COVID-19	Not impacted by COVID-19	T value	*p* value*
	Mean score (± SD)			
*Depression (PHQ-9)*				
Male	8.72 (6.42)	6.74 (6.62)	− 1.749	0.082
Female	9.14 (5.86)	6.83 (6.25)	− 5.261	< 0.001*
*Anxiety (GAD-7)*				
Male	9.36 (6.64)	7.13 (5.98)	− 1.568	0.118
Female	9.57 (6.32)	5.15 (3.80)	− 2.845	0.005*
*Resilience (CD-RISC-10)*				
Male	21.53 (8.81)	22.23 (10.39)	0.392	0.696
Female	21.40 (8.42)	21.51 (11.16)	0.094	0.925

*SD* Standard Deviation, *PHQ-9* patient health questionnaire, *GAD-7* generalized anxiety disorder-7, CD-RISC-10, Connor–Davidson Resilience Scale

*Significant at *p* < 0.05

## Discussion

### Prevalence of depressive and anxiety symptoms and COVID-19 impact

The current study suggested that the COVID-19 pandemic has had a significant impact on the mental health and well-being of this sample of university students, with four out of ten of the students self-reported moderate to severe depression (taking PHQ-9 cut-off scores of ≥ 10) and anxiety (GAD-7 cut-off scores of ≥ 10) symptoms. These levels were most prevalent among females and never-married students. The prevalence of depression in our study was higher than in what was reported in other studies [12–14, 16, 18]. In particular, among university students, several studies across the globe showed that the prevalence of depression varied, as low as 4% [41], and as high as 79.2% [42] depending on the severity and the instruments used [43–46]. In addition, according to a systemic review of published research on the mental health of young adults in the UAE between 2007 and 2017, prevalence scores ranged widely from 12.5 to 28.6% due to wide-ranging sample sizes [47].

The present study was implemented in the context of the COVID-19 pandemic. For that, our study observed higher mean PHQ-9 and GAD-7 scores among those participants who were impacted by COVID-19 than those who were categorized as not impacted. These results imply that the COVID-19 pandemic might have intensified the negative mental health impact on this sample of university students. Our findings were further supported by the results of a recent study that used PHQ-9 and the GAD-7 scales to evaluate a sample of university students during the COVID-19 pandemic and found that depressive and anxiety symptoms were prevalent in 45.2% and 38.3% of the students [48]. During stressful situations, like this pandemic, fear and anxiety about the disease can be overwhelming and it may negatively impact the mental health of all the sectors of the population [49, 50], and students in particular [51]. Fears of infection, social distancing, vaccination drives, prolonged university closure, challenges with online learning and uncertainty over examinations all cause stress and anxiety to students worldwide [6, 36, 43].

#### The effect of socio-demographic characteristics and the COVID-19 impact on the students’ mental health

The current findings revealed that depression symptoms were more reported by females than males. As previously observed, being a female was linked to a higher risk of having elevated depressive symptoms. Gender differences in depressive symptoms are typically explained in terms of gender-role socialization processes that lead to females being more likely to adopt passive cogitative responses to negative moods [52, 53]. Besides, women are more likely to be emotionally, socially and financially disadvantaged during crisis times like COVID-19 pandemic [54, 55]. This finding is consistent with a large-scale, UAE population-based survey that found females had a greater risk for depression compared to males [56]. Moreover, the present findings agree with the results of similar studies that have investigated depression among population of neighboring Gulf countries [57–59].

This study revealed that never-married university students had significantly higher depression and anxiety symptoms than their ever-married counterparts. Research speculated that marriage has been found to be associated with better mental well-being compared to other relationship statuses [60–62]. Moreover, a study confirmed that positive family-level factors (e.g. positive parenting, healthy family functions and environment) were associated with decreased depression and anxiety [62]. The married respondents enjoyed more positive family-level factors than the respondents who were not married. The unique nature of COVID-19 which offered the reduced opportunity for social interaction in single respondents while the home-bound married respondents had a robust companionship could be one of the reasons behind such findings [63]. This finding is consistent with other research [2, 30, 47, 50, 64], however, some claimed that the strength of association between single status and depression was modified by age and gender [50, 65, 66]

Although the research on the association between depression and ethnicity is inconclusive [67–70], our findings indicate that PHQ-9 is sensitive to ethnicity/ nationality, whereas UAE-national students had higher PHQ-9 scores than non-UAE national ones. The differences in the prevalence of depression outcomes may depend on whether the studies were adjusted for other factors that might be associated with depression or not [71, 72]. Factors like sociodemographic and economic profiles should be adjusted carefully. Early research provided evidence of measurement invariance of the PHQ-9 scale regarding ethnicity, implying that the observed inequalities in depressive symptoms may not be attributed to the ethnicity factor alone [73]. Some considerations can be made based on descriptions of social and cultural norms at large. Contrary to the present findings, no significant difference was observed between Emirati and non-Emirati patients in the frequency of depressive disorders using PHQ-9 [2, 74]. Our results could reflect the need to investigate the association between nationality/ ethnicity and reporting of depression symptoms among the UAE population at large.

### Resilience scores and COVID-19

The majority of the students surveyed in the present study demonstrated low to normal levels of resilience (CD-RISC 10 cut-off score of ≥ 32). It has been observed that the levels of resilience vary widely according to the sample size and the assessment tool used [75, 76]. However, considering the current total sample mean resilience score of 21.46 (± 8.80) indicates that our sample had a lower mean score than the reported mean score of 31.8 in the general population [77] and 30.97 (± 5.46) in a specific sample during COVID-19 [76]. Furthermore, the present findings highlighted that students who were categorized as impacted by COVID-19 had significantly lower resilience levels than those who were not impacted by COVID-19. Similarly, research reported that the COVID-19 stress and fear had a significant inverse correlation with resilience and that students' academic stress is negatively related to social support and resilience [78].

The present findings also showed that the resilience mean score was higher in non-employed students than in currently employed ones. This could be directly related to the increased stress levels caused by COVID-19 at the workplace. Working students might be exposed to different stressors at the workplace, including COVID-19, particularly in settings that require close human contact [20]. COVID-19 pandemic implied increased demand at the workplace in regards to the online work, travel restrictions, testing, sanitization, and vaccination drives. The UAE government applied strict work safety guidelines during the pandemic [79]. As a result, extended online working hours, added to the college's academic expectations, higher risk exposure to COVID-19 infection, changing work culture and balancing study and work could have contributed to reduced resilience in currently employed students in this study [80, 81].

### Strengths and limitations

The current study has many strengths. The novelty of the data that were collected primarily during the pandemic for this study cannot be argued, as this study added evidence to the pool of research on the mental health impact of the pandemic among a sample of university students in the UAE. The use of validated psychometric scales allows us to presume that the levels of depression and anxiety reported in this sample significantly exceeded the previously reported numbers for similar samples and could be related to COVID-19 pandemic. In addition, assessment of the demographic variables allows us to report on groups which appear to be at greatest mental health burden, and suggest a role for future interventions.

However, this study has similar limitations to other cross-sectional self-reported surveys that investigate sensitive mental health issues. First, the results represent the views of a single university student population in the UAE, that may limit the generalizability of the results. As potential participants were selected by a convenience sampling, non-random selection of the sample may limit the generalizability of this study. Another limitation may arise as there may have been a relevant difference between the students who chose to participate in the study and those who did not. It was also possible that the social desirability bias might have led some students to respond to survey items in ways that they believed were the most socially desirable [82]. Some responses also might have been influenced by confidentiality concerns as study was conducted by faculty members. Hence the above reasons might lead to some students answering in ways that they believed were the most socially desirable. Instead, it is possible that students with depression and anxiety symptoms were more willing to answer as a result of their fears about their studies.

Moreover, as the study experienced relatively high non-response rate and missing data, bias may have been introduced. However, neither of these factors should affect the attitude of those students who responded for the survey. In addition, the data was adequality managed at statistical handling to address the true values and impacts of the measured variables. Females were overrepresented in this study as in many other university settings in the UAE, which may affect the observed prevalence of depressive and anxiety in this sample. Lastly, the cross-sectional design makes it difficult to have causal relationships.

## Conclusions

The results of this study revealed that the COVID-19 pandemic has negatively impacted the mental health of this sample of university students in terms of depression and anxiety. Based on PHQ-9 and GAD-7 cut-off scores, prevalence estimates highlight that moderate to severe depression as well as anxiety symptoms were self-identified by four out of ten of the sampled students. The COVID-19 pandemic was remarkably linked to significantly higher depression and anxiety symptoms among this sample. The assessment of demographic variables revealed that differences based on gender, marital status and nationality affected the mental health of this sample and suggest a role for future interventions. This study also showed that only one in ten of the students revealed high resilience levels, however, differences in the mean CD-RISC-10 scores by COVID-19 impact were not statistically significant. In contrast, the students who were not affected by COVID-19 had a lower level of resilience. The results also revealed no significant differences in anxiety, depression, and resilience levels by gender, except when COVID-19 impact was taken as an interaction term, which further emphasize the negative impact of COVID-19 on students’ mental health.

As for the policy implications, the application of the validated PHQ-9 and GAD-7 scales are recommended as initial screening tools, however, detected cases should be later assessed using more comprehensive instruments. Besides, mental healthcare providers should offer continuous monitoring of the psychological status of university students, in particular for the vulnerable groups, and provide the required mental health support at the university setting. Strategies for could focus on increasing the availability of mental health support interventions. The results of this study highlight the importance of developing a university culture in which students could have an opportunity to communicate their mental health concerns in confidential and comfortable ways. Hotline and virtual consultations could be introduced to ensure the students confidentially and privacy. Though a huge information on students' mental health has been gathered since March 2020, research on the psychological and behavioral effects of lockdowns should still be done when the epidemic ends. Further research can include follow-ups of this sample and similar samples from various colleges and university students to allow accurate detection of the true impact of the COVID-19 pandemic on this targeted population.

## Supplementary Information

**Additional file 1.** The interaction between the effects of COVID-19 impact, marital status and nationality groups on mean scores of PHQ 9, GAD-7, and CD-RISC 10 psychometric scales.

## Data Availability

The datasets generated and analyzed during the current study are not publicly available because the data analysis is ongoing to study variables other than covered in this study. The data that supports the findings of this study are available upon request, but restrictions apply to the availability of these data. Data are however available from the authors upon reasonable request and with permission of the Higher Colleges of Technology.

## Abbreviations

ANOVA: One-way analysis of variance
CD-RISC-10: Connor–Davidson Resilience Scale
COVID-19: Coronavirus disease 2019
HCT: Higher Colleges of Technology
GAD-7: Generalized anxiety disorder
PHQ-9: Patient health questionnaire
UAE: United Arab Emirates
