# Supplementary material for: Impact of COVID-19 pandemic on the mental health of university students in the United Arab Emirates: a cross-sectional study
Source: BMC Psychol. 2022 Dec 16;10:312. doi: 10.1186/s40359-022-00986-3 (PMC9756732; doi:10.1186/s40359-022-00986-3)
Supplement: Supplementary file 1 — Additional file 1. The interaction between the effects of COVID-19 impact, marital status and nationality groups on mean scores of PHQ 9, GAD-7, and CD-RISC 10 psychometric scales. [file 40359_2022_986_MOESM1_ESM.docx]

The interaction between the effects of COVID-19 impact and marital status and nationality group on mean scores of PHQ 9, GAD-7, and CD-RISC 10 psychometric scales were non-statistically significant (Appendix 1).

| **Table 1:Tests of Between-Subjects Effects of the COVID-19 impact and Nationality Group on mean GAD-7** | | | | | | |
| --- | --- | --- | --- | --- | --- | --- |
| ***Dependent Variable:Mean_GAD_7*** | | | | | | |
| Source | | Type III Sum of Squares | df | Mean Square | F | Sig. |
| Intercept | Hypothesis | 9384.992 | 1 | 9384.992 | 51.599 | .054 |
|  | Error | 234.509 | 1.289 | 181.885^a^ |  |  |
| Nationality Group | Hypothesis | 81.232 | 1 | 81.232 | 2.230 | .136 |
|  | Error | 28709.277 | 788 | 36.433^b^ |  |  |
| COVID-19 Impact | Hypothesis | 396.222 | 1 | 396.222 | 10.875 | .001 |
|  | Error | 28709.277 | 788 | 36.433^b^ |  |  |
| a. .404 MS(COVID-19 Impact) + .596 MS(Error) | | | | | | |
| b. MS(Error) | | | | | | |

| **Expected Mean Squares^a,b^** | | | |
| --- | --- | --- | --- |
| Source | Variance Component | | |
|  | Var(COVID-19 Impact) | Var(Error) | Quadratic Term |
| Intercept | 64.486 | 1.000 | Intercept |
| Nationality Group | .000 | 1.000 | nationality |
| COVID-19 Impact | 159.512 | 1.000 |  |
| Error | .000 | 1.000 |  |
| a. For each source, the expected mean square equals the sum of the coefficients in the cells times the variance components, plus a quadratic term involving effects in the Quadratic Term cell. | | | |
| b. Expected Mean Squares are based on the Type III Sums of Squares. | | | |

| **Table 2:Tests of Between-Subjects Effects of the COVID-19 impact and Marital status on mean GAD-7** | | | | | | | | | |
| --- | --- | --- | --- | --- | --- | --- | --- | --- | --- |
| Dependent Variable:Mean_GAD_7 | | | | | | | | | |
| Source | | Type III Sum of Squares | | | df | Mean Square | | F | Sig. |
| Intercept | Hypothesis | 5827.446 | | | 1 | 5827.446 | | 50.490 | .026 |
|  | Error | 205.835 | | | 1.783 | 115.418^a^ | |  |  |
| Marital status | Hypothesis | 215.580 | | | 1 | 215.580 | | 5.945 | .015 |
|  | Error | 28574.928 | | | 788 | 36.263^b^ | |  |  |
| COVID-19 Impact | Hypothesis | 431.320 | | | 1 | 431.320 | | 11.894 | .001 |
|  | Error | 28574.928 | | | 788 | 36.263^b^ | |  |  |
| a. .200 MS(COVID-19 Impact) + .800 MS(Error) | | | | | | | | | |
| b. MS(Error) | | | | | | | | | |
| **Expected Mean Squares^a,b^** | | | | | | |  |  |  |
| Source | Variance Component | | | | | |  |  |  |
|  | COVID-19 Impact | | Var(Error) | Quadratic Term | | |  |  |  |
| Intercept | 31.859 | | 1.000 | Intercept | | |  |  |  |
| Marital status | .000 | | 1.000 | Marital status | | |  |  |  |
| COVID-19 Impact | 159.005 | | 1.000 |  | | |  |  |  |
| Error | .000 | | 1.000 |  | | |  |  |  |
| a. For each source, the expected mean square equals the sum of the coefficients in the cells times the variance components, plus a quadratic term involving effects in the Quadratic Term cell. | | | | | | |  |  |  |
| b. Expected Mean Squares are based on the Type III Sums of Squares. | | | | | | |  |  |  |

| **Table 3:Test of Between Subjects Effects of the COVID-19 impact and Nationality Group on mean PHQ-9** | | | | | | | | | |
| --- | --- | --- | --- | --- | --- | --- | --- | --- | --- |
| **Dependent Variable:Mean_PHQ_9** | | | | | | | | | |
| Source | | Type III Sum of Squares | | | df | Mean Square | | F | Sig. |
| Intercept | Hypothesis | 10109.556 | | | 1 | 10109.556 | | 22.043 | .115 |
|  | Error | 508.121 | | | 1.108 | 458.632^a^ | |  |  |
| **Nationality Group** | Hypothesis | 295.073 | | | 1 | 295.073 | | 7.664 | .006 |
|  | Error | 30531.650 | | | 793 | 38.501^b^ | |  |  |
| COVID-19 Impact | Hypothesis | 1075.783 | | | 1 | 1075.783 | | 27.941 | .000 |
|  | Error | 30531.650 | | | 793 | 38.501^b^ | |  |  |
| a. .405 MS(COVID-19 Impact) + .595 MS(Error) | | | | | | | | | |
| b. MS(Error) | | | | | | | | | |
| **Expected Mean Squares^a,b^** | | | | | | |  |  |  |
| Source | Variance Component | | | | | |  |  |  |
|  | Var(COVID-19 Impact) | | Var(Error) | Quadratic Term | | |  |  |  |
| Intercept | 64.658 | | 1.000 | Intercept | | |  |  |  |
| nationality | .000 | | 1.000 | nationality | | |  |  |  |
| COVID-19 Impact | 159.638 | | 1.000 |  | | |  |  |  |
| Error | .000 | | 1.000 |  | | |  |  |  |
| a. For each source, the expected mean square equals the sum of the coefficients in the cells times the variance components, plus a quadratic term involving effects in the Quadratic Term cell. | | | | | | |  |  |  |
| b. Expected Mean Squares are based on the Type III Sums of Squares. | | | | | | |  |  |  |

| **Table 4: Tests of Between-Subjects Effects of the COVID-19 impact and Marital Status on mean PHQ-9** | | | | | | | | | |
| --- | --- | --- | --- | --- | --- | --- | --- | --- | --- |
| Dependent Variable:Mean_PHQ_9 | | | | | | | | | |
| Source | | Type III Sum of Squares | | | df | Mean Square | | F | Sig. |
| Intercept | Hypothesis | 6220.507 | | | 1 | 6220.507 | | 23.857 | .088 |
|  | Error | 335.064 | | | 1.285 | 260.740^a^ | |  |  |
| Marital status | Hypothesis | 392.524 | | | 1 | 392.524 | | 10.228 | .001 |
|  | Error | 30434.198 | | | 793 | 38.379^b^ | |  |  |
| COVID-19 Impact | Hypothesis | 1154.469 | | | 1 | 1154.469 | | 30.081 | .000 |
|  | Error | 30434.198 | | | 793 | 38.379^b^ | |  |  |
| a. .199 MS(COVID-19 Impact) + .801 MS(Error) | | | | | | | | | |
| b. MS(Error) | | | | | | | | | |
| **Expected Mean Squares^a,b^** | | | | | | |  |  |  |
| Source | Variance Component | | | | | |  |  |  |
|  | Var(COVID-19 Impact) | | Var(Error) | Quadratic Term | | |  |  |  |
| Intercept | 31.711 | | 1.000 | Intercept | | |  |  |  |
| Marital status | .000 | | 1.000 | Marital status | | |  |  |  |
| COVID-19 Impact | 159.164 | | 1.000 |  | | |  |  |  |
| Error | .000 | | 1.000 |  | | |  |  |  |
| a. For each source, the expected mean square equals the sum of the coefficients in the cells times the variance components, plus a quadratic term involving effects in the Quadratic Term cell. | | | | | | |  |  |  |
| b. Expected Mean Squares are based on the Type III Sums of Squares. | | | | | | |  |  |  |

| **Table 5: Tests of Between-Subjects Effects of the COVID-19 impact and Nationality Group on mean CDRISC-10** | | | | | | |
| --- | --- | --- | --- | --- | --- | --- |
| \|  \| \| \| \| \| \| \| \| --- \| --- \| --- \| --- \| --- \| --- \| --- \| \| Dependent Variable:Mean_CDRISC-10 \| \| \| \| \| \| \| \| Source \| \| Type III Sum of Squares \| df \| Mean Square \| F \| Sig. \| \| Intercept \| Hypothesis \| 29330.928 \| 1 \| 29330.928 \| 402.960 \| .000 \| \| Error \| 32777.819 \| 511.421 \| 64.092^a^ \|  \|  \| \| **Nationality Group** \| Hypothesis \| .741 \| 1 \| .741 \| .010 \| .900 \| \| Error \| 40519.266 \| 659 \| 77.727^b^ \|  \|  \| \| COVID-19 Impact \| Hypothesis \| 8.815 \| 1 \| 8.815 \| .113 \| .722 \| \| Error \| 40519.266 \| 659 \| 77.727^b^ \|  \|  \| \| a. .198 MS(COVID-19 Impact) + .802 MS(Error) \| \| \| \| \| \| \| \| b. MS(Error) \| \| \| \| \| \| \|  \| **Expected Mean Squares^a,b^** \| \| \| \| \| --- \| --- \| --- \| --- \| \| Source \| Variance Component \| \| \| \| Var(COVID-19 Impact) \| Var(Error) \| Quadratic Term \| \| Intercept \| 29.417 \| 1.000 \| Intercept \| \| **Nationality Group** \| .000 \| 1.000 \| Nationality \| \| COVID-19 Impact \| 188.781 \| 1.000 \|  \| \| Error \| .000 \| 1.000 \|  \| \| a. For each source, the expected mean square equals the sum of the coefficients in the cells times the variance components, plus a quadratic term involving effects in the Quadratic Term cell. \| \| \| \| \| b. Expected Mean Squares are based on the Type III Sums of Squares. \| \| \| \| | | | | | | |
| **Table 6:Tests of Between-Subjects Effects of the COVID-19 impact and Marital Status on mean CDRISC-10** | | | | | | |
|  | | | | | | |
| Dependent Variable: Mean CDRISC-10 | | | | | | |
| Source | | Type III Sum of Squares | df | Mean Square | F | Sig. |
| Intercept | Hypothesis | 29030.928 | 1 | 29030.928 | 452.960 | .000 |
|  | Error | 32777.819 | 511.421 | 64.092^a^ |  |  |
| Marital status | Hypothesis | .741 | 1 | .741 | .010 | .922 |
|  | Error | 60549.269 | 779 | 77.727^b^ |  |  |
| COVID-19 Impact | Hypothesis | 8.815 | 1 | 8.815 | .113 | .736 |
|  | Error | 60549.269 | 779 | 77.727^b^ |  |  |
| a. .198 MS(COVID-19 Impact) + .802 MS(Error) | | | | | | |
| b. MS(Error) | | | | | | |

| **Expected Mean Squares^a,b^** | | | |
| --- | --- | --- | --- |
| Source | Variance Component | | |
|  | Var (COVID-19 Impact) | Var (Error) | Quadratic Term |
| Intercept | 31.418 | 1.000 | Intercept |
| Marital status | .000 | 1.000 | Marital status |
| COVID-19 Impact | 158.787 | 1.000 |  |
| Error | .000 | 1.000 |  |
| a. For each source, the expected mean square equals the sum of the coefficients in the cells times the variance components, plus a quadratic term involving effects in the Quadratic Term cell. | | | |
| b. Expected Mean Squares are based on the Type III Sums of Squares. | | | |
